# Effects of long-term feeding of rapeseed meal on skeletal muscle transcriptome, production efficiency and meat quality traits in Norwegian Landrace growing-finishing pigs

**DOI:** 10.1371/journal.pone.0220441

**Published:** 2019-08-07

**Authors:** Adrijana Skugor, Nils Petter Kjos, Arvind Y. M. Sundaram, Liv Torunn Mydland, Ragnhild Ånestad, Anne-Helene Tauson, Margareth Øverland

**Affiliations:** 1 Department of Animal and Aquacultural Sciences, Faculty of Biosciences, Norwegian University of Life Sciences, Aas, Norway; 2 Department of Medical Genetics, Oslo University Hospital, Oslo, Norway; 3 Department of Veterinary and Animal Sciences, Faculty of Health and Medical Sciences, University of Copenhagen, Denmark; INIA, SPAIN

## Abstract

This study was performed to investigate the effects of dietary inclusion of 20% rapeseed meal (RSM) as an alternative to soybean meal (SBM) in a three-month feeding experiment with growing finishing pigs. Dietary alteration affected growth performance, several carcass traits and transcriptional responses in the skeletal muscle, but did not affect measured meat quality traits. In general, pigs fed the RSM test diet exhibited reduced growth performance compared to pigs on SBM control diet. Significant transcriptional changes in the skeletal muscle of growing pigs fed RSM diet were likely the consequence of an increased amount of fiber and higher polyunsaturated fatty acids, and presence of bioactive phytochemicals, such as glucosinolates. RNAseq pipeline using Tophat2-Cuffdiff identified 57 upregulated and 63 downregulated genes in RSM compared to SBM pigs. Significantly enriched among downregulated pathways was *p53–mediated signalling* involved in cellular proliferation, while activation of negative growth regulators (*IER5*, *KLF10*, *BTG2*, *KLF11*, *RETREG1*, *PRUNE2*) in RSM fed pigs provided further evidence for reduced proliferation and increased cellular death, in accordance with the observed reduction in performance traits. Upregulation of well-known metabolic controllers (*PDK4*, *UCP3*, *ESRRG* and *ESRRB*), involved in energy homeostasis (glucose and lipid metabolism, and mitochondrial function), suggested less available energy and nutrients in RSM pigs. Furthermore, several genes supported more pronounced proteolysis (*ABTB1*, *OTUD1*, *PADI2*, *SPP1*) and reduced protein synthesis (*THBS1*, *HSF4*, *AP1S2*) in RSM muscle tissue. In parallel, higher levels of *NR4A3*, *PDK4* and *FGF21*, and a drop in *adropin*, *ELOVL6* and *CIDEC/FSP27* indicated increased lipolysis and fatty acid oxidation, reflective of lower dressing percentage. Finally, pigs exposed to RSM showed greater expression level of genes responsive to oxidative stress, indicated by upregulation of *GPX1*, *GPX2*, and *TXNI*P.

## Introduction

European pig production, especially in the Nordic countries, is to a large extent based on imported protein feedstuffs such as soybean meal (SBM). To improve sustainability and self-sufficiency of pig production, alternative indigenous protein feedstuffs are needed. Rapeseed is a viable alternative crop that can be grown in cold climates, and its cultivation has increased during recent years due to a growing demand for oil for biofuel production [1]. Rapeseed meal (RSM) is a co-product in oil and biofuel production, it is abundant and cheap and can serve as an alternative to SBM in diets for monogastric animals. Rapeseed meal has been used in animal diets over an extended time period, but due to a high fiber content, and anti-nutritional factors such as glucosinolates, sinapine, tannins, and erucic acid, the inclusion of RSM in pig diets usually has been kept low [2, 3]. Nevertheless, beneficial effects of RSM dietary fiber on intestinal health and overall well-being of pigs have been demonstrated [4–6].

When aiming at increasing the sustainability of pig production, feed efficiency (FE) is an essential factor both for reducing feed costs and environmental impact. Modern pig genotypes have mostly been selected for growth performance and FE under intensive feeding conditions, and Noblet et al. [7] showed that within a pig line, there was individual variation in the ability to digest organic matter, nitrogen and energy when growing finishing pigs were fed high fiber diets. The Norwegian Landrace pig used in this study is an example of such fast growing, highly efficient breed [8]. Yet, identifying the best genotypes that perform well when fed high fiber diets will be of considerable value for further improvements in sustainable pig production. For better utilization of local fiber-rich feedstuffs, more knowledge on the molecular mechanisms causing growth performance differences under those dietary regimes is required. A recent study showed that Norwegian Landrace piglets fed high dietary levels of RSM perform quite differently due to large individual variations in utilization of crude protein (CP) and neutral detergent fiber (NDF), supporting the existence of superior genotypes within the same breed that are better at digesting CP and NDF [9].

Genetic background has a large influence on animal performance, therefore, a synergy between genetics and nutrition is needed to facilitate our understanding of what constitutes optimal responses to fiber-rich diets. Only recently, it became possible to combine high-throughput omics technologies with nutrition to better understand complex biology behind economically important traits. Novel network-based approaches are being used to discover and expand gene networks associated with particular phenotypes. Recently, Vincent et al. [10] linked differences in performance between pigs selected for improved efficiency and gene expression variations in muscle tissue related to protein synthesis and mitochondrial energy metabolism. Similarly, Jing et al. [11] performed transcriptome analysis of mRNA and miRNA in skeletal muscle of Yorkshire boars and found association between gene networks controlling mitochondrial energy metabolism and muscle growth and different FE phenotypes. Another recent work compared muscle transcriptome of different pig genotypes and identified genes and regulatory networks associated with growth, fatness and metabolism [12].

The present study was conducted to evaluate the influence of two different diets: a typical commercial-like diet containing imported SBM as a control, and a locally produced diet with 20% inclusion of RSM, on animal performance, carcass and meat quality traits. The second objective was to characterize diet effects on gene expression profiles in skeletal muscle (*Longissimus dorsi*) and to identify molecular players with the largest impact on observed phenotypic differences. Hence, we employed an integrated approach combining RNAseq technology and quantitative real-time PCR (qRT-PCR) to investigate effects of a dietary alteration on global transcriptome changes in the muscle.

## Materials and methods

### Ethics statement

The experiment was conducted at the Center for Animal Research, Norwegian University of Life Sciences, Aas, Norway and the experimental protocol for the study was approved by the Norwegian Food Safety Authority (ID: 8217). All animals were cared for according to laws and regulations controlling experiments with live animals in Norway (the Animal Protection Act of December 20th, 1974, and the Animal Protection Ordinance concerning experiments with animals of January 15th, 1996).

### Animals, allotment and housing

A total of 84 purebred Norwegian Landrace pigs from 16 litters were used. Average initial weight was 24.9 kg ± 1.98 standard deviation (SD) and average final weight was 109.7 kg ± 5.44 SD. The experiment was conducted as a randomized complete block design. Pigs were blocked by litter and sex, and allotted by initial weight to two dietary treatments with 42 replicates per treatment with equal number of males and females. The experimental period lasted on average 88 days. At feeding time, each pig was restrained in an individual feeding stall until the feed was consumed in order to obtain individual feed intake. Thus, each pig was the experimental unit. The experiment was split into a growing period from start until 60 kg live weight, a finishing period from 60 kg live weight until slaughter, and the overall period. Pigs were housed in an environmentally controlled barn with partially slotted concrete floor. Fourteen 8.2 m^2^ pens designed for individual feeding were used. Due to the experimental goals, no bedding material was used, the pens were cleaned regularly, and they were equipped with rubber mats and activity enrichment tools. Average ambient daily temperature was 18.2°C (range 15.9–20.5°C). The clinical health status of the pigs was monitored daily during the feeding time. Overall, all animals were healthy throughout the experimental period and no mortalities were recorded.

### Diets and feeding

The dietary treatments were: 1) a control diet based on barley, wheat, oat and soybean meal (SBM-diet), and 2) an experimental diet based on barley, wheat, oat and 20% inclusion of commercial expeller pressed rapeseed (Mestilla UAB, Klaipeda, Lithuania) (RSM-diet). Levels of standardized ileal digestible (SID) lysine on net energy (NE) basis was adjusted to be the same in both diets by using crystalline amino acids. The diets were also designed to be isonitrogenous and isoenergetic and to contain equal levels of methionine + cysteine, and threonine. The diets were pelleted with a 3-mm die and produced by the Center for Feed Technology (FôrTek) at the Norwegian University of Life Sciences. The content of digestible lysine, threonine, methionine and cystine of the ingredients was estimated using analysed values, multiplied by the standardized ileal digestibility coefficients for nitrogen and amino acids [13]. Diets were formulated to meet or exceed the requirements for indispensable amino acids and all other nutrients [14]. A cumulative feed sample from each dietary treatment was taken for chemical analysis. Composition and analyses of diets are shown in Table 1.

**Table 1:** Ingredient and chemical composition (g kg^-1^, unless otherwise stated) of diets based on soybean meal (SBM) and rapeseed meal (RSM).

	Diet	
*Ingredients*	SBM	RSM
Barley	380.2	340.4
Wheat	240.0	233.4
Oats	140.0	140.0
Soybean meal (SBM) (45% CP)	150.0	0.0
Rapeseed meal (RSM)	0.0	200.0
Rendered fat (tallow)	50.4	50.0
Limestone	11.3	8.0
Monocalcium phosphate	16.4	16.4
Salt	4.0	4.0
L-lysine^.^ HCl (98%)	2.9	3.8
Threonine	1.5	1.5
DL-methionine	0.9	0.0
Tryptophan	0.1	0.2
Premix^1^	2.2	2.2
Yttrium oxide	0.1	0.1
*Calculated contents*, *g kg*^*-1*^		
Net energy, MJ kg^-1^	9.3	9.2
SID^2^ lysine	8.2	8.2
SID methionine + cysteine	4.9	5.0
SID threonine	5.4	5.4
SID tryptophan	1.6	1.6
Calcium	8.7	8.5
ATTD^3^ phosphorus	3.9	4.3
*Analysed content*, *g kg*^*-1*^		
DM	907.0	881.0
Gross energy, MJ kg^-1^	17.6	16.6
Crude protein	147.0	142.0
Crude fat	63.0	43.0
Ash	54.0	50.0
Neutral detergent fiber (NDF)	144.0	159.0
Acid detergent fiber (ADF)	43.0	61.0
Calcium	9.5	9.6
Total phosphorus	8.3	8.5
Lysine	9.0	8.8
Threonine	7.2	7.3
Methionine	2.7	2.4
Cysteine	2.6	2.8
Tryptophan Total glucosinolates, mmol kg^-1^	2.0 -	1.9 2.3

^1^Provided per kilogram of feed: 105 mg of Zn (ZnO); 75 mg of Fe (FeSO_4_. H_2_0); 60 mg of Mn (MnO); 15 mg of Cu (CuSO_4_ x 5H_2_O); 0.75 mg of I (Ca(IO_3_)_2_; 0.3 mg of Se (Na_2_SeO_3_); 9000 IU of vitamin A; 1125 IU of cholecalciferol; 112.5 mg of dl-a-tocopheryl acetate; 2.25 mg of menadione; 5.625 mg of riboflavin, 18.73 mg of d-pantothenic acid; 22.5 mg of cyanocobalamine; 22.5 mg of niacin; 0.225 mg of biotin; 1.69 mg of folic acid; 364 mg of choline.

^2^ Standardized ileal digestible

^3^ Apparent total tract digestible.

All pigs were individually fed twice per day according to a semi-ad libitum feeding regime providing a moderate feeding intensity during the growing period followed by an increasing feeding intensity during the finishing period [15]. Feed refusals for each pig were recorded and subtracted from the total feed intake. All pigs were given free access to water from nipple drinkers. Water was also provided directly in the trough during meals. Feed consumption and pigs’ weight were recorded weekly to determine average daily gain (ADG), average daily feed intake (ADFI), and feed conversion ratio (FCR).

### Slaughter procedure and tissue sampling

Pigs were slaughtered at the Center for Animal Research using a mobile slaughterhouse (Mobilslakt AS) under the supervision of the Norwegian Food Safety Authority. Pigs were fixed and anesthetized using head-to-heart electrical immobilization followed by exsanguination. Samples of the muscle (*L*. *dorsi*), liver, ileum and colon tissues were snap frozen in liquid nitrogen for gene expression analyses.

### Chemical analyses

Samples of dietary ingredients and the two diets were analysed for CP by Kjeldahl-N x 6.25 [16], fat using ASE 350 Accelerated Solvent Extractor, dry matter (DM) by drying to constant weight at 104°C [16], ash by incineration at 550°C [16], acid detergent fiber (ADF) and NDF using a fiber analyzer system (Ankom200, ANKOM Technologies, Fairport, NY, USA) with filter bags (Ankom F58, ANKOM Technologies). Gross energy (GE) content was determined by a Parr 1281 Adiabatic Bomb Calorimeter (Parr Instruments, Moline, IL, USA) according to [17]. Phosphorus and calcium content of the diets were analysed by atomic absorption spectrophotometry according to methods described by [18]. Analysis of amino acids in the diets was carried out according to [16] using Biochrom 30 Amino Acid Analyzer. Tryptophan in the diets was determined according to [16] using high-performance liquid chromatography system (Dionex UltiMate 3000, Dionex Softron GmbH, Germering, Germany) equipped with fluorescent detector (Shimadzu RF- 535, Shimadzu Corp, Kyoto, Japan). Glucosinolate level in the experimental RSM-diet was determined by Eurofins Food & Feed Testing Norway AS (Kambo, Moss, Norway) using liquid chromatography coupled with diode array detector- method (LC-DAD).

### Carcass characteristics

Dressing percentage was determined by the following equation: (hot carcass weight/final live weight) x 100. Live weight was monitored at the time of slaughter. Carcass lean percentage was determined commercially on the slaughter line using a GP7Q pistol (Hennessy System Ltd, Auckland, New Zealand) to measure the depth of the *L*. *dorsi* and the backfat thickness at two sites (behind the last rib, 6 cm from the midline) [15]. The prediction of carcass lean percentage was done according to an equation from the Norwegian Meat Research Centre, Oslo, Norway.

Forty-one pigs (20 from SBM and 21 from RSM group) were selected for carcass and meat quality assessment at Animalia–The Norwegian Meat and Poultry Research Centre. Backfat thickness and backfat+loin thickness including loin and fat were measured behind the last rib, 6 cm from the midline. Total fat was measured according to the method described by O’Farell et al. [19]. The carcasses were subjected to commercial cutting procedure to determine primal cut yields (backpart, midpart and frontpart), and to measure percent carcass lean and percent carcass fat. In addition, muscle pH and meat colour measures were determined from the *L*. *dorsi* muscle. Meat colour was measured after one hour blooming at 4°C using a Minolta CR400 (Minolta Co. Ltd., Osaka, Japan). Three measurements were taken from each muscle, and mean values for CIELAB L*, a*, b*, Hue and Chroma (saturation) were calculated. Values for Hue was calculated as Hue = tan^-1^ (b*/a*), and Chroma as Croma = “squareroot”(A*^2^ +b*^2^). Drip loss was measured in the *L*. *dorsi* and the loin, according to the EZ-driploss metod [20]. Collagen, fat, protein and water content in the *L*. *dorsi* was analysed using a FOSS FoodScan [21].

### RNA extraction

Total RNA from muscle, liver, ileum and colon was extracted from 6 SBM and 6 RSM pigs using TRIzol protocol (Invitrogen) followed by RNeasy Plus Mini protocol (Qiagen). After the first washing step, on-column DNAse treatment was performed using PureLink DNase kit (Invitrogen). RNA purity and quality was measured using NanoDrop 8000 spectrophotometer (Thermo Fisher Scientific, Wilmington, USA) and Agilent 2100 Bioanalyzer (Agilent Technologies, Waldbronn, Germany). Only high quality (RIN ≥ 7) samples were sent for sequencing at the Norwegian Sequencing Centre (http://www.sequencing.uio.no) and were used for validation of sequencing data by qRT-PCR. Six pigs from each dietary treatment were selected for RNA sequencing based on their FCR (three pigs with FCR higher than average and three pigs with FCR lower than average), while aiming at equal gender distribution. Pigs that were selected for sequencing originated from different litters in order to minimize the effect of single genotypes on the outcome of this study.

### Library construction and RNA sequencing

In total 48 RNA sequencing libraries were prepared with TruSeq stranded mRNA prep (Illumina, San Diego, USA) using unique barcodes and 150 bp paired end sequencing was performed across 4 lanes in HiSeq 4000 System (Illumina, San Diego, USA). Of those 48 libraries, 12 libraries contained muscle samples and the remaining 36 consisted of liver, ileum and colon samples, which will be analysed and presented in another study (data not shown). Profiling of transcriptomic responses from liver and gut tissues will provide additional understanding of effects of RSM-based diets on development and function of those organs in growing pigs. In this study, only data from 12 muscle samples was analysed and presented. Sequence data was handled using RTA v 2.7.6 and demultiplexed and converted into fastq files employing the unique barcodes using bcl2fastq v2.18.0.12.

Raw data from four lanes for each sample were concatenated together before proceeding with the analysis. Sequenced data were pre-processed by trimming/removing reads containing adapter sequences, low quality reads and PhiX genome (Illumina spike-in) using BBDuK v34.56 part of BBtools software package [22].

### Data pre-processing and differential expression analysis

Cleaned data was aligned against the *Sus scrofa*11.1 transcriptome and genome (Genbank assembly accession GCA_000003025.6) using tophat v2 [23] using—library-type fr-firststrand—no-mixed—no-novel-junc as options. Average insert fragment size for each sample was calculated by aligning 1 million cleaned paired end reads against the *Sus scrofa* cDNA using bowtie2 V2.2.3 [24] and Picard v1.112 CollectInsertSizeMetrics tool and were provided as parameters for tophat2 alignment. Differential expression of known genes (ensembl release 95) was calculated using the tophat2 aligned bam files in cuffdiff v2.2.1 using default parameters and visualised using cummeRbund v2.18.0 package [25] in R.

### cDNA synthesis and validation by qRT-PCR

To validate RNAseq data, the expression levels of *activating transcription factor 3* (*ATF3*), *thioredoxin-interacting protein* (*TXNIP*), *mitochondrial uncoupling protein 3* (*UCP3*), *parvalbumin* (*PVALB*), and *myostatin* (*MSTN*) were measured by qRT-PCR. Primers were designed using the PRIMER3 program (http://frodo.wi.mit.edu) and their full list is given in S1 Table. Prior to cDNA synthesis all samples were normalized to 300 ng/μL. cDNA synthesis was performed using AffinityScript QPCR cDNA Synthesis kit (Agilent Technologies). The qRT- PCR was performed in a total volume of 20 μL using 10 μL LightCycler 480 SYBR Green I Master, 2 μL primers, 3 μL Milli-Q water and 5 μL cDNA diluted 1:50. The PCR conditions were: 95°C for 10 min, 95°C for 10 sec, 60–64°C for 10 sec depending on the primers, 72°C for 10 sec, in a total of 40 cycles. Samples were analysed using LightCycler 480 System (Roche Diagnostics, Mannheim, Germany). All reactions were performed in duplicates and gene expression levels were quantified relative to the expression of *glyceraldehyde-3-phosphate dehydrogenase* (*GAPDH*) and *β-actin* (*ACTB*) using a mean –ΔΔCt value.

### Statistical and functional annotation analyses

Statistical analyses on animal performance and carcass characteristics were performed using the GLM procedure of SAS [26] for a complete randomized block design with individual pig as the experimental unit. Results are presented as the least square mean for each treatment, and variance is expressed as standard error of the mean (SEM). Means were separated according to the LSmeans procedure. Significant difference among treatments was shown at p<0.05.

Heatmap showing the log2(fpkm +1) values of differentially expressed genes (DEGs) was constructed based on Euclidean correlation and average linkage clustering in software Multi Experimental Viewer (MeV) (http://www.mev.tm4.org). For the functional interpretation of transcriptomic data, the identified pig DEGs were converted to human orthologs using the g:Orth tool in the g:Profiler web server [27] and then used for Gene Ontology (GO), Kyoto Encyclopedia of Genes and Genomes (KEGG) [28] and Reactome [29] enrichment analyses. To distinguish significant results from random matches, the Benjamini-Hochberg FDR method was chosen and p< = 0.05 was set as a cutoff.

## Results

### Growth performance

During the growing (p = 0.119), finishing (p = 0.002) and overall (p = 0.004) period, ADFI was lower in pigs fed the RSM diet than those fed the SBM diet. Pigs fed the RSM diet also had a lower ADG during the growing (p = 0.002), finishing (p = 0.002) and overall period (p = 0.001). The FCR was poorer in pigs fed the RSM diet in the growing (p = 0.007) and in the overall period (p = 0.012), whereas there were no differences in FCR between groups in the finishing period (p = 0.223). Similarly, the NE intake per kg gain was higher in RSM pigs during the growing and overall periods, while there was no difference between treatment groups during the finishing period (Table 2).

**Table 2:** Growth performance and carcass traits of growing-finishing pigs fed diets based on soybean meal (SBM) or rapeseed meal (RSM).

	Diet		SEM	*p*-value; effect of diet
	SBM	RSM		
Number of pigs	42	42		
*Live weight*, *kg*				
Initial	29.2	28.8	0.19	0.139
End of growing period	64.4	62.2	0.46	0.002
At slaughter	112.2	107.5	0.66	0.001
Days in experiment	88.1	88.1	0.45	0.777
*Growing period*				
ADG^1^, g	837.6	795.2	9.16	0.002
ADFI^2^, g	1707.0	1678.0	13.00	0.119
FCR^3^, g feed/g gain	2.0	2.1	0.02	0.007
NE:gain, MJ/kg gain	19.0	19.5	0.17	0.017
*Finishing period*				
ADG, g	1039.4	986.9	11.04	0.002
ADFI, g	2557.0	2470.0	17.70	0.002
FCR, g feed/g gain	2.5	2.5	0.02	0.223
NE:gain, MJ/kg gain	22.9	23.2	0.21	0.390
*Overall period*				
ADG, g	942.9	895.0	7.69	0.001
ADFI, g	2151.0	2088.0	13.40	0.004
FCR, g feed/g gain	2.29	2.34	0.01	0.012
NE:gain, MJ/kg gain	21.2	21.6	0.12	0.047
*Carcass traits*^4^				
Carcass weight, kg	78.7	73.8	0.51	0.001
Dressing percentage	70.1	68.7	0.26	0.001
Carcass lean percentage	61.9	62.1	0.25	0.845
Kg feed: kg carcass	2.4	2.5	0.01	0.001

^1^Average daily gain

^2^Average daily feed intake

^3^Feed conversion ratio

^4^ SBM diet: N = 41 and RSM diet: N = 42

### Carcass and meat quality traits

Carcasses of RSM pigs were lighter than those of SBM pigs (p = 0.001), dressing percentage was lower (p = 0.001), but carcass lean percentage was similar among dietary treatments (p = 0.845). Feed intake per kg carcass was higher in RSM than SBM pigs (p = 0.001) (Table 2). The carcass quality traits reported in Table 3 were generally very similar among dietary treatments. However, the backfat+loin thickness was significantly (p = <0.031) smaller in RSM than SBM carcasses. There were also tendencies for differences in the conformation of the carcasses with the backpart making up similar amounts in the two groups while the midpart was slightly smaller (p = 0.097) and the frontpart slightly larger (p = 0.059) in the RSM carcasses.

**Table 3:** Carcass and meat quality traits of pigs fed diets based on either soybean meal (SBM) or rapeseed meal (RSM).

	Diet		SEM	*p*-value; effect of diet
	SBM	RSM		
Number of pigs^1^	20	21		
Carcass weight, kg	76.6	74.8	1.01	0.199
Carcass lean percent, GP7	62.3	62.0	0.73	0.780
Backfat (mm) ^2^	13.8	12.7	0.58	0.188
Backfat+loin thickness (mm) ^2^	54.1	50.1	1.24	0.031
Loin (LD), fat (mm)	10.0	9.2	0.85	0.493
Total carcass fat (%)^3^	18.0	17.7	0.93	0.847
*Primal cuts method*				
Percent carcass lean	63.0	63.0	0.73	0.968
Percent carcass fat	19.5	19.5	0.93	0.951
Backpart, %	34.9	34.8	0.30	0.899
Midpart, %	35.9	35.3	0.25	0.097
Frontpart, %	29.2	29.9	0.24	0.059
Drip loss, loin, %	5.4	5.2	0.41	0.702
Drip loss, belly, %	4.7	4.7	0.54	0.948
pH, LD-muscle	5.5	5.4	0.02	0.473
Collagen, Foodscan (%)	1.0	1.1	0.03	0.039
Fat, Foodscan (%)	1.2	1.2	0.05	0.606
Protein, Foodscan (%)	23.3	23.3	0.14	0.967
Water, Foodscan (%)	74.4	74.3	0.11	0.604
Minolta Chrome	9.1	9.8	0.35	0.170
Minolta Hue	20.6	21.8	1.17	0.424

^1^ Data from 20 and 21 pigs belonging to control and RSM group respectively were used for the primal cut evaluation.

^2^ Measurements of backfat and backfat+loin thickness were performed according to [15].

^3^ Measured according to [19].

Meat quality traits were measured on 20 SBM and 21 RSM carcasses, and there were generally no differences between treatment groups, the only significant difference being a slightly higher content of collagen in RSM carcasses (p = 0.039) (Table 3).

### Transcriptomic analysis

RNA sequencing was performed to identify changes in muscle gene expression profiles of 12 Norwegian Landrace pigs belonging to the two groups (SBM and RSM). On average sequencing resulted in 30 M 150bp paired end reads per sample, of which more than 98% were retained after pre-processing for alignment and further analyses (S2 Table). Up to 71% of these cleaned reads aligned to the reference genome/transcriptome using the tophat2 pipeline and nearly 99% of these were of concordant alignment. The sequence reads have been submitted to the NCBI Sequence Read Archive (SRA) under accession number PRJNA506343.

**Fig 1:**
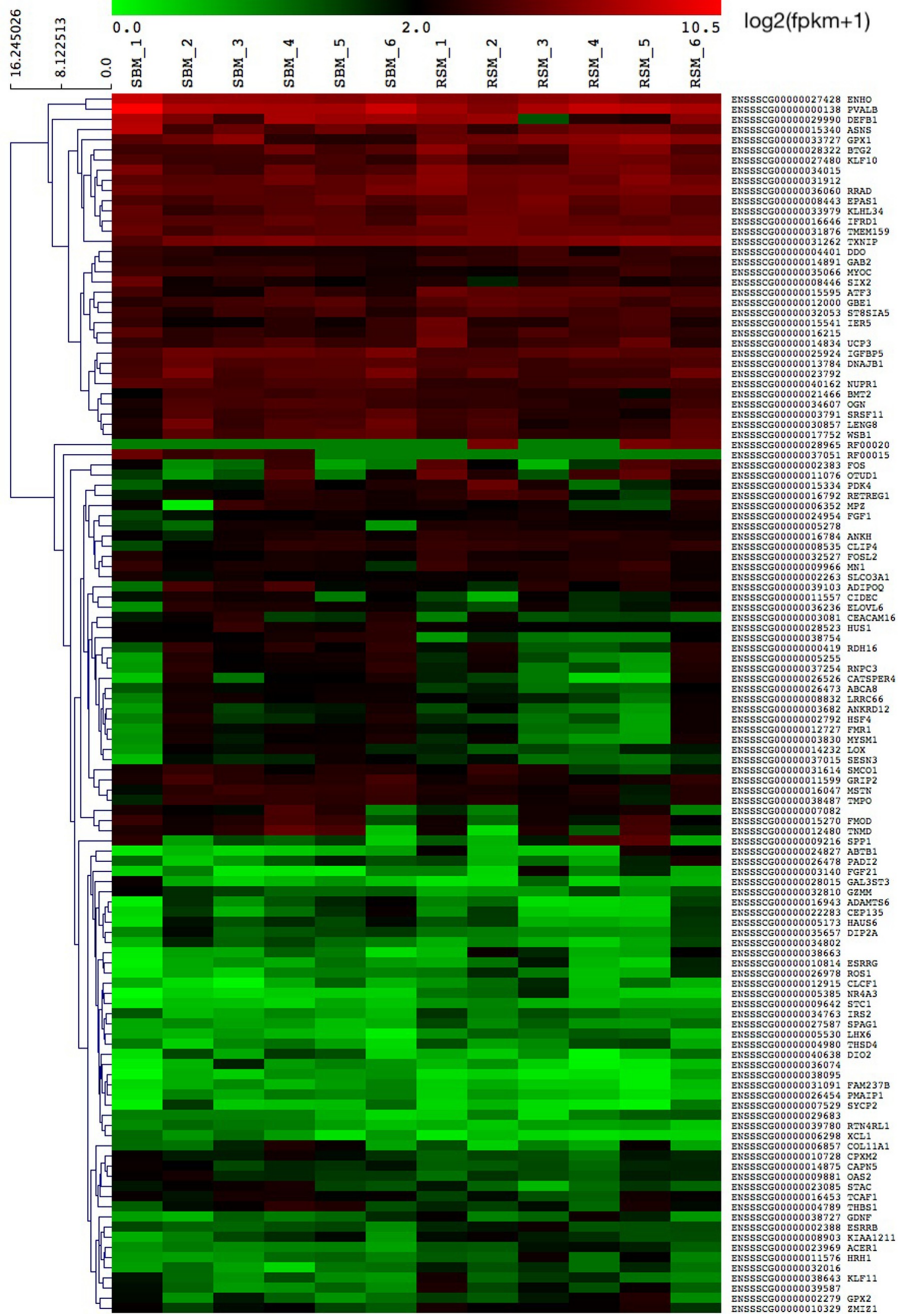
Heat map of gene expression profiles in control SBM and experimental RSM group. Heatmap is showing the log2(fpkm +1) values. The genes are clustered using Euclidean correlation and average linkage clustering in MeV.

The expression of 25,878 genes was tested in the muscle tissue of pigs subjected to RNAseq. The criteria for DEGs was selected by comparing the test RSM group to the control SBM, with the log2-fold change = >0.5; p< = 0.05 and q< = 0.05 adjusted for FDR, which resulted in 57 upregulated and 63 downregulated DEGs. Previous studies investigating muscle gene expression patterns in pigs reported that alterations in expression profiles could be small [30] [31], hence, in this study, all transcripts with p< = 0.05 and q< = 0.05 were considered DEGs. Volcano plot for the DEGs is shown in S1 Fig. Heat map of gene expression profiles in control SBM and experimental RSM group is shown in Fig 1 and the complete info of all identified DEGs is shown in S3 Table.

**Fig 2:**
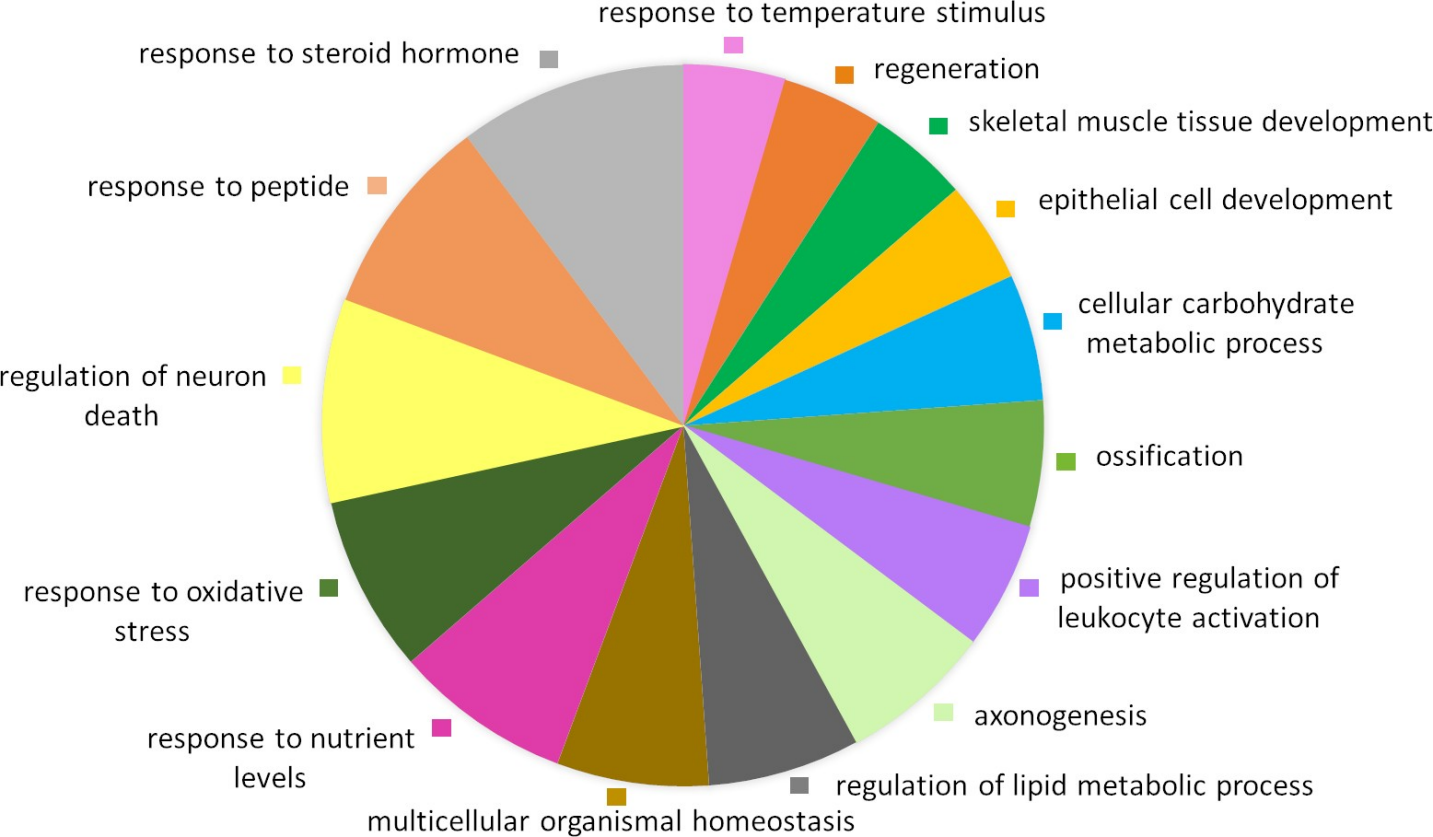
Gene Ontology analysis showing significantly enriched biological processes among upregulated DEGs in the muscle of RSM pigs in comparison to SBM pigs. Biological processes represented by less than 4 genes were excluded.

Due to the incomplete annotation of the reference pig genome (*Sus scrofa*11.1), the DEGs were converted to human orthologs and the web server g:Profiler was used for functional analyses of enriched biological processes and pathways. The analysis of 49 upregulated genes that entered GO analysis revealed 21 enriched biological processes, most of which were related to lipid and carbohydrate metabolic process, skeletal muscle tissue development and function, response to nutrient levels and energy homeostasis and oxidative stress. Reactome identified *nuclear receptor transcription* and *RET signaling pathways* as enriched among the upregulated genes. A pie chart with an overview of enriched biological processes represented by at least four or more upregulated genes is shown in Fig 2. The group of 63 downregulated genes, of which 60 were used for analysis, were enriched for 2 biological processes, skeletal muscle adaptation and cell aggregation. Enrichment analysis of biological pathways among downregulated genes revealed two enriched pathways, *p53 signaling pathway* and *diseases of glycosylation* (pie chart not shown). The complete list of enriched GO processes and KEGG and Reactome pathways with corresponding p-values is provided in S4 Table.

### Differentially expressed genes between RSM and SBM fed pigs

A number of DEGs orchestrating muscle metabolism and development were regulated: a growth factor *FGF21* that functions as a key metabolic regulator induced by different kinds of stress; *FOS* encoding leucine zipper protein implicated in skeletal muscle signal transduction, cell proliferation and differentiation; *LHX6* developmental protein implicated in differentiation, neurogenesis and transcription regulation; *IFRD1/TIS7* controlling growth, differentiation and muscle tissue regeneration; *ESRRG* and *ESRRB* expressed in tissues with high metabolic demand like skeletal muscle where they play important roles in fiber-type determination, muscle vascularization, oxidative and lipid metabolism; *FGF1* implicated in myogenesis and muscle regeneration and negative regulator of skeletal muscle growth *MSTN*. In addition, dietary treatment affected the levels of transcripts mediating calcium signaling and muscle contraction, with *STC1* and *RRAD* showing increased expression, while *PVALB* and *STAC* exhibited lower mRNA levels (Table 4).

**Table 4:** Differentially expressed genes in the muscle of the RSM pigs in comparison to the SBM pigs. Data are log2-fold change (FC).

Muscle metabolism and development	Short gene name	log2-FC	Functional description	References
*Fibroblast growth factor 21*	*FGF21*	3.52	energy homeostasis, mitochondrial function, oxidative stress	[32]
*Fos proto-oncogene*	*FOS*	1.70	muscle metabolism, signaling, apoptosis	[33]
*LIM homeobox 6*	*LHX6*	1.20	transcription regulator, differentiation, neurogenesis, development	[34]
*Estrogen related receptor gamma*	*ESRRG*	0.91	transcription regulator, muscle development, oxidative metabolism	[35] [36]
*Estrogen related receptor beta*	*ESRRB*	0.72	transcription regulator, muscle development, oxidative metabolism	[37]
*Fibroblast growth factor 1*	*FGF1*	0.60	growth factor, muscle development	[38]
*Interferon related developmental regulator 1*	*IFRD1/TIS7*	0.57	muscle cell differentiation, skeletal muscle regeneration	[39]
*Myostatin*	*MSTN*	-0.84	regulator of myogenesis, muscle weight	[40]
**Calcium and potassium signaling**				
*Stanniocalcin 1*	*STC1*	0.89	calcium ion homeostasis	[41]
*Ras related glycolysis inhibitor and calcium channel regulator*	*RRAD*	0.63	calcium and insulin signaling, muscle fiber type determination	[42]
*SH3 and cysteine rich domain*	*STAC*	-1.14	muscle contraction, ion transport	[43]
*Parvalbumin*	*PVALB*	-0.98	muscle protein, calcium ion binding, contraction	[33]
**Negative regulation of proliferation, apoptosis, stress response**				
*Ankyrin repeat and BTB domain containing 1*	*ABTB1/BPOZ*	2.59	growth-suppressive signaling, proteolysis	[44]
*Activating transcription factor 3*	*ATF3*	1.54	transcription regulator, stress response	[45] [46]
*Immediate early response 5*	*IER5*	1.20	transcription regulator, growth-suppressive signaling	[47]
*BTG anti-proliferation factor 2*	*BTG2*	1.16	growth-suppressive signaling	[48]
*Kruppel like factor 10*	*KLF10/TIEG1*	1.06	proliferation repressor, transcription repressor, muscle weight	[49] [50]
*Reticulophagy regulator 1*	*RETREG1*	0.95	autophagy, ER-phagy, apoptosis	[51]
*Protein prune homolog 2*	*PRUNE2*	0.84	pro-apoptotic	[52]
*Kruppel like factor 11*	*KLF11/TIEG3*	0.72	anti-proliferative, apoptosis	[53]
*Asparagine synthetase (glutamine-hydrolyzing)*	*ASNS*	-1.03	cell cycle, cell growth	[54]
*Centrosomal protein 135*	*CEP135*	-0.78	centriole formation, cell cycle	[55]
*Insulin like growth factor binding protein 5*	*IGFBP5*	-0.68	myogenesis activator, growth	[56]
*Nuclear protein 1*, *transcriptional regulator*	*NUPR1*	-0.68	stress response, negative regulation of cell cycle	[57]
*Structural maintenance of chromosomes 5*	*SMC5*	-0.63	cell cycle, cell division, DNA damage, stress response	[58] [59]
**Positive regulation of proliferation**				
*Histamine receptor H1*	*HRH1*	1.09	proliferation, angiogenesis, development, growth	[60] [61]
*Glial cell derived neurotrophic factor*	*GDNF*	0.94	growth factor, positive regulation of proliferation	[62]
*ROS proto-oncogene 1*. *receptor tyrosine kinase*	*ROS1*	0.94	differentiation, proliferation, vascular remodeling, stress response	[63]
*Zinc finger MIZ-type containing 1*	*ZMIZ1*	0.71	artery development, vasculogenesis, fibroblast proliferation	[64] [65]
*GRB2 associated binding protein 2*	*GAB2*	0.57	signalling, positive regulation of cell proliferation, neurogenesis	[66]
*Phorbol-12-myristate-13-acetate-induced protein 1*	*PMAIP1/NOXA*	-0.88	DNA damage, apoptosis, response to oxidative stress	[67]
*Base methyltransferase of 25S rRNA 2 homolog*	*SAMTOR*	-0.68	mTORC1 signaling, cell growth	[68]
*HAUS augmin like complex subunit 6*	*HAUS6*	-0.66	cell cycle, cell division	[69]
**Protein metabolism/protein turnover**				
*OTU deubiquitinase 1*	*OTUD1*	1.69	proteolysis, protein transport	[70]
*Phenylalanine—tRNA ligase beta subunit-like*	*FARSB*	1.06	protein biosynthesis	
*Peptidyl arginine deiminase 2*	*PADI2*	0.73	citrullination of vimentin, degradation	[71]
*Adaptor related protein complex 1 subunit sigma 2*	*AP1S2*	-1.34	protein transport	[72]
*Heat shock factor protein 4*	*HSF4*	-0.90	unfolded protein response, stress response	[73]
*Fragile X mental retardation 1*	*FMR1*	-0.71	translation repressor, proteasomal protein catabolism	[74]
*WD repeat and SOCS box containing 1*	*WSB1/SWIP1*	-0.63	protein ubiquitination, protein modification	[75]
*Calpain 5*	*CAPN5*	-0.61	proteolysis, neuronal differentiation, response to stress	[76]
**Carbohydrate, lipid and energy metabolism**				
*Nuclear receptor subfamily 4 group A member 3*	*NR4A3*	2.03	metabolic function, energy homeostasis, oxidative metabolism	[77] [78]
*Alkaline ceramidase 1*	*ACER1*	1.18	ceramide degradation, sphingosine-1-phosphate signaling	[79]
*Pyruvate dehydrogenase kinase 4*	*PDK4*	1.11	glucose and fatty acid metabolism	[80] [81]
*Insulin receptor substrate 2*	*IRS2*	0.76	insulin signaling in skeletal muscle	[82]
*1*.*4-alpha-glucan branching enzyme 1*	*GBE1*	0.61	glycogen metabolism	[83]
*ST8 alpha-N-acetyl-neuraminide alpha-2*.*8-sialyltransferase 5*	*ST8SIA5*	0.57	sphingolipid biosynthesis, protein glycosylation	[84]
*Retinol dehydrogenase 16 (all-trans)*	*RDH16*	-0.84	electron transport, vitamin A metabolism, lipid metabolism	[85]
*Energy homeostasis associated*	*ENHO/adropin*	-0.79	glucose homeostasis, lipid and energy metabolism	[86] [87]
*Adiponectin*	*ADIPOQ*	-0.75	adipokine, β-oxidation	[88]
*Cell death inducing DFFA like effector c*	*CIDEC/FSP27*	-0.68	lipid droplet organization, lypolysis	[89]
*ELOVL fatty acid elongase 6*	*ELOVL6*	-0.63	fatty acid elongation, lipogenesis	[90]
**Biotransformation/xenobiotic metabolism**				
*Glutathione peroxidase 1*	*GPX1*	1.30	antioxidant enzyme	[91] [92]
*Glutathione peroxidase 2*	*GPX2*	0.93	antioxidant enzyme	[91] [93]
*Uncoupling protein 3*	*UCP3*	0.78	response to ROS, mitochondrial FA transport	[94]
*Thioredoxin interacting protein*	*TXNIP*	0.62	response to ROS, energy metabolism	[95] [96]
*Endothelial PAS domain protein 1*	*EPAS1*	0.62	oxygen sensor, response to hypoxia, proliferation, angiogenesis	[97]
*ATP-binding cassette sub-family A member 8*	*ABCA8*	-0.88	detoxification, transport	[98]
*Thrombospondin 1*	*THBS1/TSP1*	-0.62	cell adhesion, angiogenesis, oxidative stress, unfolded protein response	[99]
*Sestrin 3*	*SESN3*	-0.55	metabolic regulator, response to oxidative stress	[100]
**Extra cellular matrix and cell adhesion**				
*Secreted phosphoprotein 1*	*SPP1/OPN*	1.79	cytokine, signaling, ECM remodelling; muscular dystrophy	[101] [102] [103]
*Thrombospondin type 1 domain containing 4*	*THSD4*	0.77	matrix assembly	[104]
*Otoraplin*	*OTOR*	-1.27	connective tissue development	[105]
*Carcinoembryonic antigen related cell adhesion molecule 16*	*CEACAM16*	-1.20	cell adhesion, cell growth, angiogenesis	[106]
*Tenomodulin*	*TNMD*	-1.13	angiogenesis inhibitor, marker of tendons and ligaments	[107]
*ADAM metallopeptidase with thrombospondin type 1 motif 6*	*ADAMTS6*	-1.05	ECM proteolysis, biosynthesis of collagen	[108]
*Collagen type XI alpha 1 chain*	*COL11A1*	-0.99	connective tissue, ECM	[109]
*Fibromodulin*	*FMOD*	-0.81	ECM, collagen fibril organization	[110]
*Osteoglycin*	*OGN*	-0.78	ECM, collagen fibrillogenesis, growth factor	[111]
**Cytoskeleton and cell motility**				
*CAP-Gly domain containing linker protein family member 4*	*CLIP4*	0.72	microtubule binding, intracellular transport	[112]
*Tubulin alpha-1D chain*	*TUBA1D*	0.59	assembly of the cytoskeleton	[113]
*Myocilin*	*MYOC*	-0.56	cytoskeletal function	[114]

One of the most highly expressed transcripts in the RSM fed pigs was the mRNA coding for *ABTB1* elongation factor involved in PTEN growth-suppressive signaling and protein degradation via ubiquitin- proteasome pathway. Similarly, the amount of *OTUD1* mRNA was more than 3 fold greater in RSM pigs implying higher level of proteasomal activity. Further, the RSM pigs exhibited higher levels of transcripts implicated in growth-suppressive signaling and negative regulation of proliferation and apoptosis: *IER5*, *BTG2*, *KLF10*, *KLF11*, *RETREG1* and *PRUNE2*. The cell cycle regulators *ASNS*, *CEP135*, *SMC5* and *NUPR1* and myogenesis promoter *IGFBP5*, were all downregulated in RSM group, further suggesting reduced cell proliferation. Nevertheless, observed regulation of transcripts involved in growth suppression and apoptosis in RSM fed pigs was accompanied with downregulation of negative regulators of skeletal muscle mass *MSTN*, *CAPN5* and *SAMTOR* possibly promoting protein synthesis and muscle growth. In addition, RSM fed pigs had higher levels of several positive regulators of proliferation including *HRH1*, *GDNF*, *ZMIZ1* and *GAB2*.

*IRS2* transcript encoding a cytoplasmic signaling molecule regulating insulin-mediated glucose transport was upregulated in RSM compared to SBM pigs, while expression of *ENHO* involved in insulin signaling, metabolic adaptation to fasting and glucose homeostasis decreased. Transcript involved in glycogen metabolism, *GBE1*, exhibited higher expression in pigs fed RSM compared to the SBM diet.

Majority of lipid metabolism regulators were downregulated by RSM dietary treatment including *RDH16*, *ADIPOQ*, *FSP27* and *ELOVL6*, while *ACER1* and *ST8SIA5* were found upregulated. Expression of *NR4A3* transcript involved in the regulation of glucose and fatty acid (FA) utilization genes in skeletal muscle and modulation of feeding behavior and energy balance was more than 4 fold higher in pigs receiving the RSM diet. Moreover, the fiber containing RSM diet increased expression of *FGF21*, *PDK4*, *ESRRB* and *ESRRG*, which are all known metabolic regulators activated under the conditions of deprived energy or oxidative stress (Table 4).

Dietary treatment affected a number of genes with functions in cellular detoxification pathways and response to reactive oxygen species (ROS) (Table 4). Interestingly, several transcripts with beneficial roles in reducing oxidative stress and enhancing free radical scavenging were more abundant in RSM pigs: *GPX1*, *GPX2*, *TXNIP*, and *UCP3*. The RSM pigs also had higher levels of an adaptive-response gene *ATF3*, involved in ER stress response, and *EPAS1*, which is responsive to hypoxic stress. *ABCA8*, downregulated by RSM, is a member of the xenobiotic transporter ABC-subfamily, which facilitates cholesterol efflux and mediates transport of xenobiotics, while *THBS1*, also downregulated by RSM, mediates ER stress response by inducing protective antioxidants.

Two transcripts involved in cytoskeleton organization and cell motility, *CLIP4* and *TUBA1D*, were upregulated in RSM pigs, while *MYOC* exhibited lower levels compared to control. The majority of genes involved in the organization of the extra cellular matrix (ECM) and cell-matrix adhesion were reduced in RSM pigs, evidenced by the expression of *TNMD*, *CEACAM16*, *COL11A1*, *ADAMTS6*, *FMOD* and *OGN* (Table 4).

### Validation of sequencing data by quantitative RT-PCR

**Fig 3:**
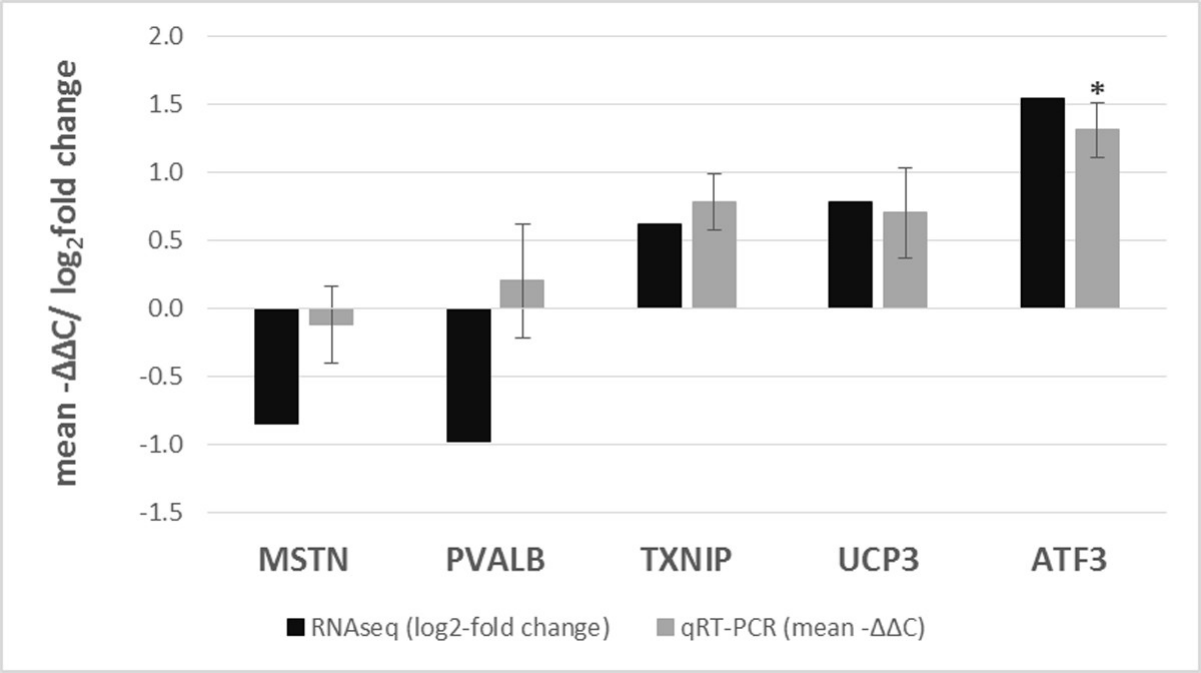
Validation of RNAseq data by qRT-PCR for 5 selected genes. RNAseq data is shown as log2-fold change, while qPCR data is shown as mean –ΔΔCt ± SD, so that they are comparable, (n = 6). All RNAseq results are significant; significant difference detected by qRT-PCR is marked with *. The significance level was set to p<0.05. The expression of selected genes was normalized to *GAPDH*- *glyceraldehyde-3-phosphate dehydrogenase* and *ACTB*- *β-actin* in the qRT-PCR analysis. *TXNIP- thioredoxin-interacting protein*; *UCP3- uncoupling protein 3 (mitochondrial*, *proton carrier)*; *ATF3- Activating transcription factor 3*; *PVALB- parvalbumin; MSTN- myostatin*.

qRT-PCR of 5 genes was performed to validate RNAseq data. The majority of genes selected for validation included those involved in the regulation of cellular redox systems (*TXNIP*, *UCP3*, *ATF3*) and energy and muscle metabolism (*PVALB* and *MSTN*). Validation revealed high correspondence between RNAseq and qRT-PCR for 4 genes, while higher number of animals are likely needed to confirm *PVALB* expression (Fig 3).

## Discussion

A number of studies show that increased use of RSM in pig diets holds great potential to improve sustainability and self-sufficiency, while simultaneously reducing environmental burden [115–117]. Here, we report effects of a three-month dietary replacement of SBM with RSM on growth performance, carcass and meat quality traits and skeletal muscle gene expression in growing finishing Norwegian Landrace pigs.

Despite equal calculated net energy and nitrogen contents, and balanced amino acid profiles of the two diets, feeding RSM reduced growth performance. Multiple factors in the RSM diet could contribute to this observation, including lower feed intake, higher content and composition of fiber, and content of bioactive compounds, mainly glucosinolates. Increased use of RSM in diets for pigs is limited by the properties of the low degradable fiber fraction in the RSM, which reduces the availability of energy and nutrients [118–120]. The physicochemical properties of the RSM fiber, including the swelling, water-holding capacity, solubility, degradability and fermentability, have been shown to influence pig performance by affecting feed intake, satiety, digesta passage rate, digestion efficiency and absorption of nutrients [121, 122]. Young pigs fed a similar RSM based diet, using the same RSM type (Mestilla UAB) as in the present experiment, had lower digestibility of nutrients and energy compared to the SBM based control [123]. Detailed profiling of the fiber component revealed that replacement of SBM with RSM resulted in increased content of non-starch polysaccharides, NDF, cellulose and lignin, demonstrating the presence of more indigestible fiber in the RSM compared to the SBM. A similar difference in the fiber fraction between the RSM and the SBM diet can be anticipated in the present study.

The RSM diet resulted in reduced carcass weight and dressing percentage of the pigs. The negative effect on carcass yield is commonly observed after an extended exposure of fibrous diets to pigs, which could at least partially be attributed to viscera enlargement [124]. It has also been shown that larger viscera not only reduce carcass dressing percentage, but at the same time increase energy and nutrient requirements, additionally affecting FCR [125, 126]. In a recent review, Agyekum and Nyachoti [127] provided a detailed description of how feeding fiber-rich diets to pigs leads to increase in maintenance requirements due to enlarged viscera and liver. Signaling transducers, growth factors, hormones and receptors involved in pathways that regulate muscle tissue development and metabolism were found regulated.

At slaughter, the SBM group weighed on average 5 kg more than the RSM group, a difference close to 5%. However, the lower cost of the RSM diet can at least partly compensate for reduction in performance. There were large individual differences in ADG and ADFI in both dietary treatments suggesting that performance traits are to a great extent affected by genetics. Higher levels of NDF and ADF in the RSM diet likely had the largest effect on growth performance, but anti-proliferative effects of glucosinolates could also be a contributing factor [3]. Reduced palatability of glucosinolate-rich diets with consequent negative effects on feed intake is well-documented [128–130]. The concentration of glucosinolates with 20% RSM inclusion slightly exceeded the recommended limit of 2.1 mmol/kg feed for pigs [131], and some of the observed responses could therefore be attributed to biological properties of glucosinolates. Harmful effects of glucosinolates are mainly associated with their breakdown products, goitrogenic substances, which when present in higher amounts interfere with iodine metabolism and disturb the function of the thyroid gland consequently affecting animal performance [3, 128].

DEGs significantly affected by dietary treatments were classified according to their biological function and associated pathways. In general, dietary treatments caused moderate skeletal muscle transcriptional changes. However, RNAseq profiling revealed potentially important differences that could be related to RSM-mediated reduction in growth performance and carcass traits, metabolic adaptations, and heightened response to oxidative stress.

### Regulation of cellular proliferation, ECM remodeling and muscle development

In response to the lower available energy and nutrients in the RSM diet, pigs fed this diet showed higher levels of numerous transcripts encoding factors involved in negative regulation of cell growth and apoptosis (*IER5*, *BTG2*, *KLF10/TIEG1*, *KLF11/TIEG3*, *RETREG1*, *PRUNE2*). Downregulation of three essential negative regulators of skeletal muscle mass (*MSTN*, *CAPN5* and *FMR1*) suggested that cellular growth in RSM pigs was low and did not require additional inhibitory regulation. RET signaling pathway, which is essential for development of neuronal lineages [132], was activated in RSM pigs, and likely had a role during muscle tissue remodeling, as previously described in developing zebrafish muscle [133]. RET signaling is activated by the secreted neurotrophic factor *GDNF* that modulates connections between nerve and muscle, which showed higher mRNA levels in RSM pigs [134]; [135]. *LHX6* was another transcript implied in neural and muscle development that showed the same pattern of expression as *GDNF*. Upregulated genes involved in positive regulation of proliferation (*EPAS1*, *HRH1*, *ROS1*, *ZMIZ1*) could be involved in the proliferation of cells other than skeletal muscle cells. Overall, the net effect of genes promoting apoptosis on one hand, versus those stimulating proliferation suggests reduced proliferation and tissue growth in RSM fed pigs. The use of fiber and polyunsaturated fatty acid rich diets that contain bioactive phytochemicals such as glucosinolates from cruciferous vegetables, provides a number of beneficial health effects for humans, including prevention of obesity and chemoprevention mediated by anti-proliferative, as well as anti-oxidant and anti-lipogenic properties of these diets [136].

Genes involved in pathways controlling protein breakdown and synthesis and regulation of muscle mass (*ABTB1*, *OTUD1*, *PADI2*, *SPP1*) might play a role in hypotrophy in RSM pigs. In addition to the lower availability of nutrients and energy of the RSM diet, lower feed intake in this group could also affect expression of these genes. Two of the most highly expressed transcripts, *ABTB1* and *OTUD1*, suggested increased rate of protein breakdown via ubiquitin proteasome system. Similarly, RSM group showed high expression of *SPP1*, whose elevated levels were reported in muscle dystrophy accompanied by loss of muscle mass [102]. Gene profiles with roles in skeletal muscle development may be associated with accelerated proteolysis, but they may also play homeostatic functions during normal muscle growth, which requires both protein synthesis and degradation.

RNAseq analysis showed downregulation of numerous genes regulating ECM and connective tissue development in RSM pigs, including the mRNA coding for procollagen *COL11A1*, and genes important for collagen assembly and ECM structure and function (*TNMD*, *OTOR*, *CEACAM16*, *FMOD*, *ADAMTS6*, *OGN*). Signaling transducers, growth factors, hormones and receptors also likely involved in pathways that regulate muscle tissue development and metabolism were among the top regulated genes (Table 4). Upregulation of *SPP1/OPN* in RSM further supported need for tissue remodeling and reorganization of ECM [101]. Changes of ECM could affect overall tissue architecture and homeostasis [137, 138], as well as the meat quality traits [137, 139]. However, despite the tissue remodeling that occurred in response to RSM diet, no differences in meat quality traits between the two groups were observed. Slightly higher content of collagen in RSM carcasses (Table 3) can be considered negligible.

### Metabolic adaptations to RSM diet

Growing skeletal muscle has large metabolic plasticity to withstand various environmental cues, but variations in dietary composition can pose a metabolic challenge during development affecting muscle mass [140]. Studies have shown that changes in factors mediating metabolic processes contribute to the variation in FE traits [141, 142]. In our study, dietary treatment affected genes and pathways with roles in metabolic activity regulation (*FGF21*, *FGF1*, *FOS*, *PDK4*, *NR4A3*, *ESRRB*, *ESRRG* and *nuclear receptor transcription pathway*), some of which were previously reported to affect FE traits in mammals [10, 143, 144]. Molecular regulation of skeletal muscle metabolism is primarily regulated by nuclear receptors, which function as transcription factors that bind DNA and mediate gene expression regulation orchestrating FA metabolism, oxidative phosphorylation and mitochondrial biogenesis [145]. Hence, upregulation of *nuclear receptor transcription pathway* in the muscle of RSM pigs could be responsible for major differences in skeletal muscle transcription profiles between dietary treatments. In this respect, activation of nuclear receptors *ESRRB* and *ESRRG* might have stimulated the upregulation of *PDK4* expression [146], a kinase that decreases glucose utilization and increases fat metabolism in response to lower energy levels [80]. Moreover, a downregulation of *ENHO/adropin* transcript, which plays a critical role in carbohydrate and lipid metabolism and metabolic adaptation to fasting and upregulation of *IRS2*, *FGF21*, *NR4A3*, *RRAD* and *TXNIP* involved in insulin and glucose transport suggested that RSM pigs could have enhanced need for glucose uptake compared to control pigs, in order to compensate for the lower energy status. Gao et al. [87] proposed that *adropin* regulates the preference for fuel selection in skeletal muscle in the feeding and fasting cycle, and plays a role in governing glucose and lipid homeostasis. Hence, reduced levels of *adropin* mRNA in muscle of RSM fed pigs might have induced a shift to fat use rather than glucose resulting in increased FA uptake and oxidation. This was further supported by marked upregulation of *FGF21*, a key mediator of energy metabolism that activates lipolysis, increases FA oxidation and enhances glucose uptake in response to nutritional status [147] [148].

In line with this, functional annotation of upregulated DEGs highlighted three biological processes in the RSM muscle: ‘multicellular organismal homeostasis’, ‘response to nutrient levels’ and ‘energy reserve metabolic process’ (Fig 2 and S4 Table)–a set of adaptive biochemical and physiological changes to reduce metabolism and activate pathways to derive energy from stored compounds such as lipids or glycogen. Additional evidence for reduced anabolic processes in RSM fed pigs included regulation of several key genes involved in lipid catabolism and FA oxidation; lower levels of *ELOVL6* encoding the only elongase involved in *de novo* lipogenesis [149]; downregulation of *CIDEC/FSP27*, which controls triglycerides accumulation and formation of lipid droplets [89]; higher level of *UCP3* mitochondrial transporter, which enhances muscle FA oxidation [150].

In accordance to our findings, two recent studies in pigs reported that dietary supplementation of polyunsaturated fatty acids and bioactive polyphenols stimulated the expression of genes involved in lipogenesis and oxidative processes in skeletal muscle [90] [151]. Rapeseed contains high amounts of oleic, linoleic and linolenic long chain fatty acids, which also have the ability to affect expression of lipid metabolic genes [152, 153]. Recent RNAseq studies in pigs investigated effects of dietary supplementation of rapeseed and linseed oils with higher concentration of polyunsaturated fatty acids on transcription profiles in liver and muscle tissues [154] [155]. In addition to anti-adipogenic effects of dietary long chain fatty acids, addition of dietary fiber have also been reported to reduce lipogenesis in pigs [156]. In agreement with this, our RNAseq data revealed a number of genes possibly affected by different composition of dietary long chain fatty acids between the two diets (*IRS2*, *FGF21*, *FGF1*, *PDK4*, *HRH1*, *UCP3*, *NR4A3*). Genes coding for *FGF21*, *NR4A3*, *PDK4*, *UCP3* and *IRS2* that were more abundant in the RSM group, were reported to promote muscle lipid oxidation and leaner growth by regulating genes involved in mitochondrial and peroxisomal FA oxidation. *FGF21* has been described as a marker of mitochondrial dysfunction and stress, which is induced in muscle to enhance mitochondrial function and efficiency and help in stress adaptation by activating the mTOR-YY1-PGC1α pathway [32]. Similarly, *ESRRG* transcription factor which had higher expression in RSM pigs has been reported to activate oxidative potential and is considered a key regulator of oxidative metabolism that increases mitochondrial activity in skeletal muscle. Rangwala et al. [35] reported higher levels of *ESRRG* mRNA in oxidative, slow-twitch muscle fibers and low levels in glycolytic fibers. In this study, regulation of key genes associated with muscle mitochondrial activity and energy metabolism control (*FGF21*, *ESRRG*, *ESRRB*, *NR4A3*, *PDK4*) indicated a shift toward the oxidative muscle phenotype, characterized by enhanced mitochondrial function and increased fuel uptake in the skeletal muscle exposed to RSM in comparison to SBM diet.

Scheffler and Gerrard [157] showed that muscle fibers with greater oxidative capacity usually exhibit increased protein turnover which can lead to decrease in efficiency and growth potential. Jing et al. [11] reported downregulation of *ESRRB* transcript in the skeletal muscle of more efficient pigs showing a negative correlation between improved FE and muscle remodeling toward oxidative phenotype and increased mitochondrial metabolism. In view of these findings, similar adverse effects on performance traits due to increased muscle oxidative metabolism in RSM fed pigs could be anticipated.

### Response to oxidative stress

The GO analysis of upregulated genes revealed enriched biological functions associated with modulation of cellular redox state in RSM pigs, namely, response to ROS and oxidative stress (Fig 2 and S4 Table). One of the well-recognized beneficial properties of bioactive phytochemicals from RSM is their potent antioxidant activity and modulation of cellular redox state by the induction of glutathione-based Phase-2 detoxifying enzymes across vertebrates [158, 159], including fish [160]. This is in agreement with observed higher levels of transcripts involved in maintenance of antioxidant defenses and repression of ROS in the RSM group, including *GPX1* and *GPX2* from the glutathione pathway, *TXNIP* and *UCP3*.

Interestingly, several studies have reported metabolic effects of dietary phytochemicals on signaling networks controlling energy metabolism through the regulation of cellular redox state [136, 161, 162]. Lower energy level provided by the RSM diet with the resultant tissue downsizing (by increased proteolysis, decreased cellular proliferation and increased apoptosis), and metabolic adaptations (stimulation of mitochondrial biogenesis, and switch to better utilization of lipid metabolism) strongly suggested lower level of anabolic processes, and consequently lower threat of oxidative stress. Therefore, activation of Phase-2 *GPX1* and *GPX2* enzymes in RSM pigs can be considered beneficial. The findings of this study could be extrapolated to other farmed animals and humans. Functional consequences of RSM-based and similar diets need to be further investigated to ensure optimal use with no negative effects.

## Conclusion

Pigs fed a diet containing 20% RSM with higher content of fiber and bioactive phytochemicals, including glucosinolates, showed somewhat reduced growth performance and dressing percentage compared to pigs fed a control SBM diet. Yet, dietary treatments had no effect on meat quality traits.

The differential gene expression profile reflected growth performance differences between pigs receiving the two diets, revealing underlying molecular mechanisms of observed phenotypes. The analysis of global changes in expression of genes regulating protein degradation, tissue remodeling, carbohydrate and lipid metabolism, mitochondrial adaptation and ROS metabolism improved our understanding of the effects of RSM-based diets on growth performance and skeletal muscle of growing-finishing Norwegian Landrace pigs. The research will help facilitate a more sustainable Norwegian pig production characterized by increased use of local ingredients and reduced environmental impact.

## Supporting information

S1 Table Primers used for real-time quantitative PCR. (DOCX)

S2 Table RNAseq statistics. (DOCX)

S3 Table Complete list of differentially expressed genes. (XLSX)

S4 Table Complete list of enriched biological processes and pathways among up- and downregulated differentially expressed genes. (XLSX)

S1 Fig Volcano plot for differentially expressed genes. X-axis shows the fold change (log2) vs p value (-log10). Significantly differentially expressed genes (log2-fold change = > 0.5; p value < = 0.05) area marked in red.
(PNG)
