# Supplementary material for: Effects of long-term feeding of rapeseed meal on skeletal muscle transcriptome, production efficiency and meat quality traits in Norwegian Landrace growing-finishing pigs
Source: PLoS One. 2019 Aug 7;14(8):e0220441. doi: 10.1371/journal.pone.0220441 (PMC6685631; doi:10.1371/journal.pone.0220441)
Supplement: S1 Table — (DOCX) [file pone.0220441.s001.docx]

**Supplementary file S1.** Primers used for real-time quantitative PCR.

| Gene name | Gene symbol | Primers | Sequences (5´-3´) | Size (bp) | |
| --- | --- | --- | --- | --- | --- |
| *Activating transcription factor 3* | *ATF3* | Forward | ACCCCTCGAGATGTCAGTCAC | | 193 |
|  |  | Reverse | CTCCTCAATTTGGGCCTTCAGTTC | |  |
| *Thioredoxin-interacting protein* | *TXNIP* | Forward | CAGGGGAGAATGAGATGGTG | | 166 |
|  |  | Reverse | TCTTGAGTTGGCTGGCTTG | |  |
| *Mitochondrial uncoupling protein 3* | *UCP3* | Forward | ACGATGGATGCCTACAGGAC | | 195 |
|  |  | Reverse | TCCGAAGGCAGAGACAAAGT | |  |
| *Myostatin* | *MSTN* | Forward | AAAGGCCCAACTGTGGATATATCTG | | 247 |
|  |  |  |  | |  |
|  |  | Reverse | TGACCATTCTCATCTAAAGCTTTGA | |  |
|  |  |  |  | |  |
| *β-actin* | *ACTB* | Forward | CCAGGTCATCACCATCGG | | 158 |
|  |  | Reverse | CCGTGTTGGCGTAGAGGT | |  |
| *Glyceraldehyde-3-phosphate dehydrogenase* | *GAPDH* | Forward | ACACTCACTCTTCTACCTTTG | | 90 |
|  |  | Reverse | CAAATTCATTGTCGTACCAG | |  |
