# Supplementary material for: Effects of long-term feeding of rapeseed meal on skeletal muscle transcriptome, production efficiency and meat quality traits in Norwegian Landrace growing-finishing pigs
Source: PLoS One. 2019 Aug 7;14(8):e0220441. doi: 10.1371/journal.pone.0220441 (PMC6685631; doi:10.1371/journal.pone.0220441)
Supplement: S2 Table — (DOCX) [file pone.0220441.s002.docx]

**Table S2. RNA sequencing of mRNA from muscle tissue of Norwegian Landrace pigs fed either SBM or RSM diet. Reads were mapped to the pig Sscrofa11.1 genome assembly.**

|  |  | | **Tophat2 alignment overall** | **Tophat2 alignment concordant** |
| --- | --- | --- | --- | --- |
|  | **Sequenced reads** | **Cleaned reads** |  |  |
| 1-SBM | 40,187,345 | 39,656,551 | 64.5% | 64.2% |
| 2-SBM | 20,524,276 | 20,262,359 | 71.4% | 71.1% |
| 3-SBM | 25,301,601 | 24,909,193 | 71.0% | 70.7% |
| 4-SBM | 30,192,728 | 29,721,412 | 67.9% | 67.7% |
| 5-SBM | 27,944,104 | 27,510,611 | 66.8% | 66.6% |
| 6-SBM | 35,889,773 | 35,391,057 | 68.8% | 68.5% |
| 1-RSM | 20,741,994 | 20,442,783 | 66.1% | 65.9% |
| 2-RSM | 24,995,512 | 24,645,298 | 65.2% | 64.9% |
| 3-RSM | 40,440,277 | 39,823,568 | 68.1% | 67.8% |
| 4-RSM | 31,570,100 | 31,162,808 | 70.3% | 70.0% |
| 5-RSM  6-RSM | 31,216,550  31,632,527 | 30,715,455  31,156,608 | 68.9%  70.5% | 68.6%  70.1% |
|  | | | | |
