# Supplementary figures and images for: Effects of long-term feeding of rapeseed meal on skeletal muscle transcriptome, production efficiency and meat quality traits in Norwegian Landrace growing-finishing pigs
Source: PLoS One. 2019 Aug 7;14(8):e0220441. doi: 10.1371/journal.pone.0220441 (PMC6685631; doi:10.1371/journal.pone.0220441)

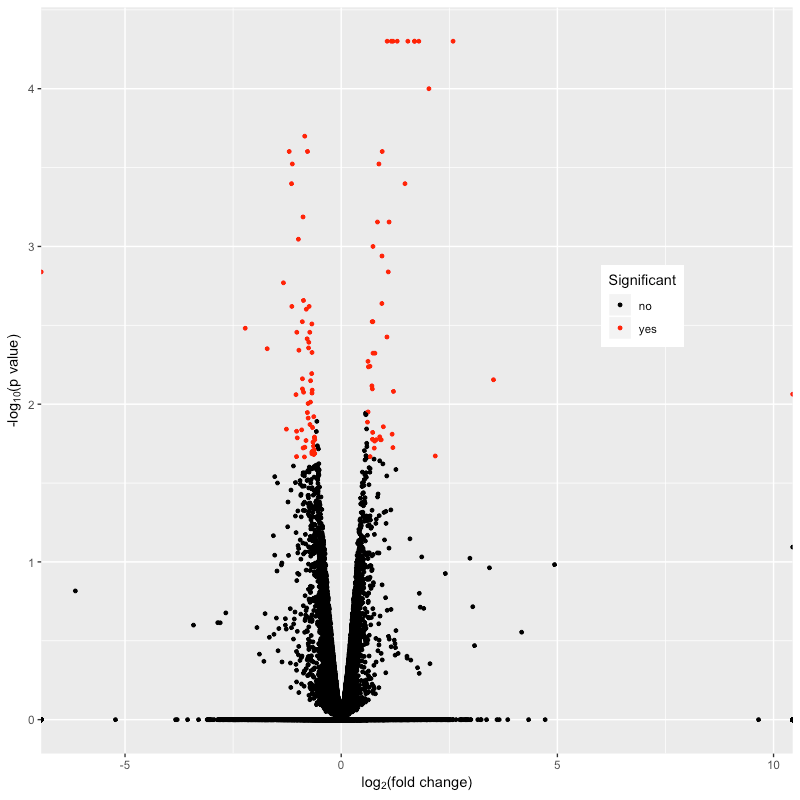

Supplement: S1 Fig — X-axis shows the fold change (log2) vs p value (-log10). Significantly differentially expressed genes (log2-fold change = > 0.5; p value < = 0.05) area marked in red. (PNG) [file pone.0220441.s005.png]
